# Seasonal Variations in the Diagnosis of Testicular Germ Cell Tumors: A National Cancer Registry Study in Austria

**DOI:** 10.3390/cancers13215377

**Published:** 2021-10-27

**Authors:** Gennadi Tulchiner, Nina Staudacher, Josef Fritz, Monika Hackl, Martin Pichler, Maximilian Seles, Shahrokh F. Shariat, David D’Andrea, Kilian Gust, Walter Albrecht, Karl Grubmüller, Stephan Madersbacher, Sebastian Graf, Lukas Lusuardi, Herbert Augustin, Andreas Berger, Wolfgang Loidl, Wolfgang Horninger, Renate Pichler

**Affiliations:** 1 Department of Urology, Medical University Innsbruck, Anichstrasse 35, 6020 Innsbruck, Austria; Gennadi.Tulchiner@i-med.ac.at (G.T.); Nina.Staudacher@i-med.ac.at (N.S.); Wolfgang.Horninger@i-med.ac.at (W.H.); 2 Department of Medical Statistics, Informatics and Health Economics, Medical University of Innsbruck, Schöpfstraße 41, 6020 Innsbruck, Austria; Josef.Fritz@i-med.ac.at; 3 Austrian National Cancer Registry, Statistics Austria, 1110 Vienna, Austria; Monika.Hackl@statistik.gv.at; 4 Research Unit of Non-Coding RNAs and Genome Editing in Cancer, Comprehensive Cancer Center Graz, Division of Clinical Oncology, Department of Internal Medicine, Medical University of Graz, 8036 Graz, Austria; martin.pichler@medunigraz.at; 5 Department of Urology, Medical University of Graz, 8036 Graz, Austria; maximilian.seles@medunigraz.at; 6 Department of Urology, Comprehensive Cancer Center, Medical University of Vienna, 1090 Vienna, Austria; shahrokh.shariat@meduniwien.ac.at (S.F.S.); david.dandrea@meduniwien.ac.at (D.D.); kilian.gust@meduniwien.ac.at (K.G.); 7 Department of Urology, Austria and Public Health Agency of Lower Austria, 2130 Mistelbach, Austria; walter.albrecht@noe-lga.at; 8 Department of Urology and Andrology, University Hospital Krems, Karl Landsteiner University of Health Sciences, 3500 Krems, Austria; karl.grubmueller@krems.lknoe.at; 9 Department of Urology and Andrology, Kaiser-Franz-Josef Spital, 1100 Vienna, Austria; stephan.madersbacher@wienkav.at; 10 Department of Urology, Johannes Kepler University Linz, 4040 Linz, Austria; Sebastian.Graf@kepleruniklinikum.at; 11 Department of Urology and Andrology, Paracelsus Medical University Salzburg, Müllner Hauptstrasse 48, 5020 Salzburg, Austria; l.lusuardi@salk.at; 12 Department of Urology, General Hospital of the City of Klagenfurt, 9020 Klagenfurt, Austria; Herbert.Augustin@kabeg.at; 13 Department of Urology, Academic Teaching Hospital Feldkirch, 6800 Feldkirch, Austria; Andreas.Berger@lkhf.at; 14 Department of Urology, Ordensklinikum Linz GmbH Elisabethinen, 4020 Linz, Austria; wolfgang.loidl@ordensklinikum.at

**Keywords:** detection, diagnosis, seasonal variation, testicular cancer, vitamin D, screening, incidence, germ cell tumors

## Abstract

**Simple Summary:**

Seasonal variations in cancer diagnosis could already be demonstrated in prostate and breast cancer. The reasons for this observed seasonal pattern are still unclear. The health care system or other determinants such as the protective function of vitamin D3 in carcinogenesis could be assumed as one explanation. Testicular germ cell tumors are the most common developed malignancy among young men. The aim of our study was to investigate, for the first time, the seasonal variations in the clinical diagnosis of testicular germ cell tumors. We have been able to confirm that the frequency of monthly newly diagnosed cases of testicular cell tumors in Austria has a strong seasonality, with a significant reduction in the tumor incidence during the summer months and an increase during the winter months.

**Abstract:**

We conducted a retrospective National Cancer Registry study in Austria to assess a possible seasonal variation in the clinical diagnosis of testicular germ cell tumors (TGCT). In total, 3615 testicular cancer diagnoses were identified during an 11-year period from 2008 to 2018. Rate ratios for the monthly number of TGCT diagnoses, as well as of seasons and half-years, were assessed using a quasi-Poisson model. We identified, for the first time, a statistically significant seasonal trend (*p* < 0.001) in the frequency of monthly newly diagnosed cases of TGCT. In detail, clear seasonal variations with a reduction in the tumor incidence during the summer months (Apr–Sep) and an increase during the winter months (Oct–Mar) were observed (*p* < 0.001). Focusing on seasonality, the incidence during the months of Oct–Dec (*p* = 0.008) and Jan–Mar (*p* < 0.001) was significantly higher compared to the months of Jul–Sep, respectively. Regarding histopathological features, there is a predominating incidence in the winter months compared to summer months, mainly concerning pure seminomas (*p* < 0.001), but not the non-seminoma or mixed TGCT groups. In conclusion, the incidence of TGCT diagnoses in Austria has a strong seasonal pattern, with the highest rate during the winter months. These findings may be explained by a delay of self-referral during the summer months. However, the hypothetical influence of vitamin D3 in testicular carcinogenesis underlying seasonal changes in TGCT diagnosis should be the focus of further research.

## 1. Introduction

Seasonal variations in the diagnosis of cancer are most pronounced in breast and prostate cancer, confirming a sharp decrease in the number of new cases during the summer months. This observed seasonal pattern presumably depends on health-care system factors, with reduced activity of screening programs during summer months and personal behaviors [1]. However, evidence supporting the protective function of vitamin D3 in carcinogenesis is mounting. Expression of the vitamin D receptor (VDR) has been shown to exhibit tumor-suppressing and anti-proliferative effects, promoting apoptosis as well as inhibiting angiogenesis [2]. Thus, cancer seasonality seems to be influenced by vitamin D3, supporting the relationship between vitamin D3 and cancer.

Testicular germ cell tumors (TGCT) are the most commonly developed malignancy among young men aged between 15–40 years, accounting for 5% of urological tumors. TGCT is characterized in 90% of cases by unilateral palpable scrotal mass, mostly detected by physical self-examination. Screening on a population basis is not recommended under current guidelines [3]. Geographic disparities in the incidence of TGCT, with the highest rate in Northern Europe, are well known but without clear explanations [4]. To our knowledge, no previous study has investigated seasonal variations in the clinical diagnosis of TGCT.

## 2. Materials and Methods

### 2.1. Patients and Data Collection

This cancer registry study was approved by the local ethics committee of the Medical University Innsbruck (study number 1055/2021). All testicular cancer diagnoses were identified via the International Statistical Classification of Diseases and Related Health Problems (ICD)-10 codes (C62.0, C62.1 and C62.9) from the Austrian National Cancer Registry (ANCR) during an 11-year period from 2008 to 2018. The ANCR, operated by Statistics Austria, the National Statistical Institution, is a population-based cancer registry covering the total Austrian population. Cancer notifications are mandatory for all hospitals based on legal regulations since 1969. The completeness of the data after diagnosis over the 5 year registration period has been estimated to be 94% [5]. ANCR data are linked with official causes of death (CoD) to ascertain death certificate only (DCO) cases and for follow-up. Data were extracted from the ANCR on 17 December 2020, follow-up was complete by 31 December 2019.

### 2.2. Statistics

For statistical analysis, the association between the monthly number of TGCT diagnoses and diagnosis month in terms of rate ratios (RRs) was assessed using a quasi-Poisson model [6], adjusted for year (to account for a potential secular trend) and using the number of days of the respective month as an offset value (to account for differences in the number of diagnoses caused by differences in length of the observation period). Although there was no evidence of overdispersion in our count data (*p* = 0.387 from the overdispersion test), we decided to use the more flexible quasi-Poisson approach, compared to the traditional Poisson model. By replacing the variable month by an indicator for season (1 for Jan–Mar, 2 for Apr–Jun, 3 for Jul–Sep and 4 for Oct–Dec), and an indicator for half-year (1 for Apr–Sep, 2 for Oct–Mar) in the quasi-Poisson model, we acquired RRs for the comparison of seasons and half-years. Subgroup analyses regarding age, tumor spread and histology were performed, and effect modification assessed via likelihood ratio tests. Finally, a year-adjusted quasi-Poisson model for the association between time of year and number of diagnoses was built using natural cubic splines with evenly spaced knots every three month. The existence of a seasonal trend was assessed by comparing the spline model vs. a base model without the variable time of year via a likelihood ratio test. All statistical tests were two-sided, and statistical significance was defined as *p* < 0.05. Statistical analyses and generation of the figure was conducted in R, version 4.0.3.

All cases with a diagnosis date on the first day of a month (i.e., Jan 1st, Feb 1st, etc.) were excluded. This was conducted to avoid statistical biases resulting from diagnoses of an unknown month for which diagnosis dates were routinely input as Jan 1st in the registry. To have a fair comparison of TGCT diagnosis across all months, we excluded also the first day of all other months to have a consistent statistical approach.

## 3. Results

### 3.1. Descriptive Characteristics

Out of a total of 4108 testicular cancer diagnoses identified via International Statistical Classification of Diseases and Related Health Problems (ICD)-10 codes (C62.0, C62.1, C62.9) from the Austrian National Cancer Registry between 2008 and 2018, we excluded 493 cases with a diagnosis date on the first day of a month, leaving 3615 cases with a mean (±SD) age of 38.6 ± 12.7 years for final analysis.

Regarding histomorphological features, 2392 of all tumors were pure seminomas with a mean (±SD) age of 41.6 ± 11.4 years, while 840 of the tumors could be identified as non-seminomas with a mean (±SD) age of 32 ± 10.9 years and 125 cases as mixed TGCT (age: 32.5 ± 10.5 years). In 258 cases, definitive histological classification was missing.

### 3.2. Significant Seasonal Patterns in the Incidence of TGCT Diagnoses

**Figure 1:**
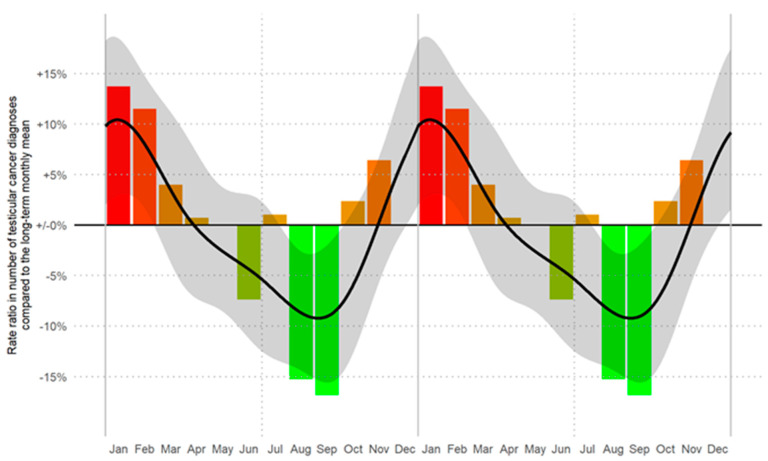
Rate ratios (RRs) of the number of TGCT diagnoses by month of diagnosis. Crude RRs (corrected for the length of the respective month) are displayed as bars. The smoothed RR curve, modelled via natural cubic splines, is overlaid in black together with 95% confidence bands (shaded). A likelihood ratio test showed a significant seasonal trend (*p* < 0.001). The horizontal reference line refers to the long-term (time period between 2008 and 2018) monthly average number of diagnoses of 27.4 cases/month (RR = 1.00). Data are displayed as a double plot for better visualization of the seasonal trend. From red to green in descending order of RRs.

We identified, for the first time, a statistically significant seasonal trend (*p* < 0.001) in the frequency of monthly newly diagnosed cases of TGCT. The incidence of new TGCT diagnoses was highest during the winter months (January to March), followed by a steady decrease in subsequent months and a renewed increase in October to December (Figure 1).

During the cold months (Oct–Mar), the incidence was 13% higher than during the warm months (Apr–Sep; *p* < 0.001). This trend was observed independently for the period 2008–2013 (11% increase) and 2014–2018 (16% increase). In detail, incidence rates increased by 22% during Jan–Mar as compared to Jul–Sep (*p* < 0.001), by 15% during Oct–Dec (*p* = 0.008), and by 9% during Apr–Jun (*p* = 0.096). Further analyses by single months and subgroups are described in Table 1. Clear seasonal variations with a reduction in the tumor incidence during the summer months and increase during the winter months were observed. In detail, the overall incidence of testicular cancer in the winter months, Oct–Mar (*n* = 1917), was significantly higher than in the summer months Apr–Sep (*n* = 1698), *p* < 0.001.

Focusing on seasonality, the incidence during the months Oct–Dec (*n* = 937, *p* = 0.008) and Jan–Mar (*n* = 980, *p* < 0.001) was significantly higher compared to the months Jul–Sep (*n* = 817), respectively. There were no obvious differences in this pattern between the observation period 2008–2013 and 2014–2018 (*p_interaction_* = 0.602), Table 1.

Focusing on pure seminomas, there is a predominating incidence in Oct–Mar (*n* = 1 302) compared to Apr–Sep (*n* = 1090), *p* < 0.001. In contrast, no differences between winter months and summer months were detected in the non-seminoma or mixed TGCT groups. In further subgroup analyses, the trend of higher incidences of new TGCT cases during winter months persisted for age and tumor spread with no effect modification present, Table 1.

### 3.3. Reproducibility of Data Generation

**Figure 2:**
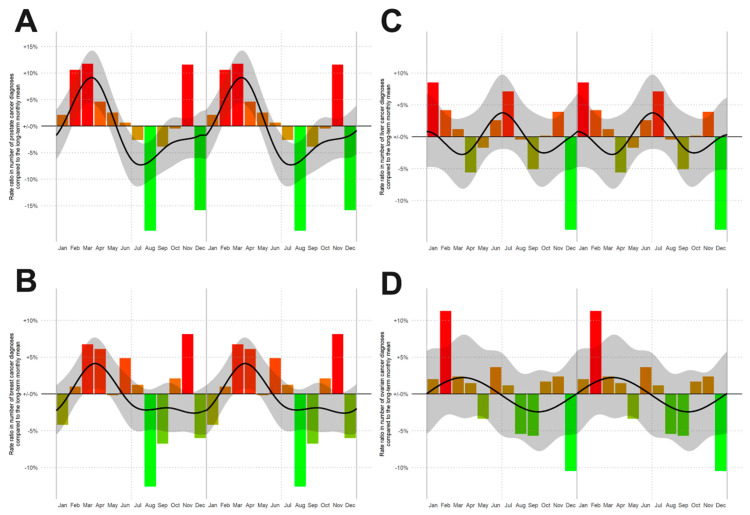
Rate ratios (RRs) of the number of new cancer diagnoses by month of diagnosis for: (**A**) prostate cancer, (**B**) breast cancer, (**C**) liver tumor and (**D**) ovarian carcinoma. From red to green in descending order of RRs. The smoothed RR curve, modelled via natural cubic splines, is overlaid in black together with 95% confidence bands (shaded).

To exclude statistical inaccuracies in data generation, we also built similar spline models for the incidence rates of other cancer entities such as liver, ovary, breast and prostate cancer, confirming seasonality of incidence rates for prostate (Figure 2A, *p* < 0.001, *n* = 48,692) and breast (Figure 2B, *p* = 0.001; *n* = 51,793) cancer, but not for liver (Figure 2C, *p* = 0.139, *n* = 9339) and ovarian cancer (Figure 2D, *p* = 0.812, *n* = 6791), (Figure 2). These findings are in line with the published data about seasonal patterns in new cancer diagnoses [1] corroborating the correct data captured in our registry study.

## 4. Discussion

Seasonal variation of cancer incidence was already described for melanomas, prostate, breast and thyroid cancer based on data generated from the Swedish Cancer Registry [1]. Moreover, there are other data supporting the evidence of temporal patterns in cancer diagnoses for hemalogic malignancies such as leukemia or lymphoma [7,8]. On the contrary to melanoma, Lambe et al. [1] confirmed a significant reduction in the numbers of diagnosed cancer cases during the summer months for prostate, thyroid and breast cancer, similar to our results observed for testicular cancer. These temporal patterns of cancer diagnoses might reflect either differences in the health care system regarding delayed cancer detection due to reduced capacity to screen and register patients, or biological phenomena [1]. However, to our knowledge, no previous study has investigated seasonal variations in the diagnosis of TGCT.

We have been able to show, for the first time, that the frequency of monthly newly diagnosed cases of testicular cancer in Austria have a strong seasonality. Considering the fact that Austria has equal access to public services with a free of charge and uniform national health care system, it is unlikely that these temporal incidence patterns can be explained by systematically delayed data reporting or data entry. Secondly, reproducibility and correctness of data generation has been confirmed by seasonal variation patterns of other cancer entities in accordance with the literature. Moreover, as there are no standardized and recommended screening programs for TGCT, our findings of decreased TGCT diagnoses during the summer months cannot be attributed to a reduced capacity of screening but are more likely to reflect a delay of self-referral. Concerning our results, in contrast to non-seminoma or mixed GCT, seasonal variations of TGCT diagnosis mainly concerned pure seminoma and localized disease. It is speculative to imagine that non-seminomas, which are usually more rapidly rising, and metastatic disease, being more characterized by symptoms, are diagnosed more in “real-time” and thus, without seasonal variations. However, a common clinical feature of TGCT, especially for organ-confined disease, is that it often presents with nonacute and mild symptoms, and thus argues in favor of a delay of self-referral. However, although its incidence has increased during recent decades [4], wide variations in mortality rates have been reported from the International Agency for Research on Cancer compiled in GLOBOCAN 2008 [9]. Whereas TGCT mortality rates are declining in industrialized countries, the mortality rates are stable or steadily increasing in countries transiting toward higher levels of development [9]. Reasons for these findings might be unequal access to specialized treatment, lack of awareness and delay of immediate diagnosis and treatment [9]. Due to the fact that its incidence has increased during previous decades, especially in industrialized countries [10], further exploration of these temporal patterns of TGCT diagnosis could be of particular interest, because of the possible negative consequences related to the seasonal delay between the first visit, referral and final diagnosis of TGCT. 

We hypothesize that another possible reason of these temporal patterns may reflect complex biological phenotypes in the likelihood of TGCT detection. Vitamin D can modulate cells of both the innate and adaptive immune system [11,12] affecting simultaneously the differentiation, growth and apoptosis of cancer cells via various mechanisms [13,14]. This immunomodulatory effect of vitamin D3 via intracellular VDR could be described on multiple immune cell lineages such as monocytes/macrophages, T cells, B cells, natural killer cells (NK) and dendritic cells (DCs) [15]. Thus, vitamin D3 is known as a regulator for the normal development and function of NK [16]. Further, a suppression of macrophages resulting in an anti-inflammatory M2 macrophage phenotype [17] as well as inhibition of differentiation and survival of DCs [18] by vitamin D was revealed. The adaptive immune system is regulated by vitamin D3 mainly by stimulating T cell differentiation and activation [19]. Promoting the proliferation and effector function of Foxp3^+^ Tregs, vitamin D3 supports the immune response suppression and mediates immune tolerance [20,21,22]. Consequently, the association between vitamin D deficiency and autoimmune disease, such as rheumatoid arthritis (RA), systemic lupus erythematosus (SLE), antiphospholipid syndrome (APS), Hashimoto’s thyroiditis (HT) and multiple sclerosis (MS) can be explained [23,24,25]. On the other hand, children with vitamin D deficiency have lower levels of naive CD4+ T cell, CD4+ T-helper and CD8+ cytotoxic T lymphocytes [26].

Several epidemiologic trials confirmed that a low vitamin D status is also a risk factor for several types of cancer such as breast cancer [27], prostate cancer [28,29] and colorectal cancer [30] suggesting that vitamin D supplementation might be cancer preventive. Nevertheless, there are still conflicting data in this field, and large randomized control trials could not corroborate these findings for the general population. Thus, a stratification into more responsive sub-populations would be helpful [14,31]. Interestingly, it has been demonstrated that vitamin D3 also regulates testicular cell proliferation and apoptosis [32,33]. Initial evidence of testicular expression of VDR has also been reported in vitro and in vivo [33]. A high prevalence of vitamin D3 deficiency has been confirmed in patients affected by TGCT and is associated with embryonal carcinoma and advanced clinical stage [34]. For seminomas higher tumor-infiltrating lymphocytes (TILs) density was associated with a lower clinical tumor stage and a lack of lymphovascular invasion at presentation [35]. Furthermore, the prognostic relevance of TIL count in seminomas regrading disease relapse is suspected [35]. Moreover, seminoma is typically more infiltrated with a higher number of T cells compared to non-seminoma [36]. In our study, there was a strong predominance of seasonal appearance for pure seminoma, but not for non-seminoma or mixed GCT. This association could possibly be linked to vitamin D promoted lymphocyte differentiation. However, further research about a link between vitamin D3 in the pathogenesis of TGCT and the observed seasonal occurrence is further needed.

Our study has some limitations that should be mentioned. It is an analysis of a national cancer registry reflecting data of only one country. However, data are of high validity to which all TGCTs are reported in Austria with a uniform national public health care system. Of course, confounding factors such as a delay of self-referral or the influence of vitamin D3 in testicular carcinogenesis could be involved in the seasonality and these findings may be disputed. Furthermore, the sun irradiation time could influence the possible vitamin D3 effect on carcinogenesis of TGCT. On the other hand, Austria is located at latitudes between 46° and 49°, where the sun exposure time should be comparable. Other factors that may affect vitamin D3 status, such as vitamin D dietary intake, obesity, alcohol consumption, smoking and socioeconomic status could not be controlled for in this study [37]. It is also conceivable that the lower diagnostic intensity during the summer reflects a reduced capacity to evaluate patients with suspected cancers. Evaluation of seasonal patterns of TGCT diagnosis across multiple countries at once, for example adding other cancer registry data, would make this type of study clearer and might confirm if there is any consistency of our preliminary findings.

## 5. Conclusions

In summary, we observed that the registered incidence of TGCT diagnoses in Austria has a strong seasonal pattern, with the highest rate during the winter months (January to March), followed by a steady decrease in subsequent months and a renewed increase in October to December. These findings may be explained by a delay of self-referral during the summer. However, in order to understand other possible influences such as vitamin D3 in testicular carcinogenesis underlying seasonal changes in TGCT diagnosis, replicating these analyses in the northern European populations and in countries that do not have significant seasonal variations in sunlight would be the next step of research.

## Figures and Tables

**Table 1:** Quasi-Poisson regression models for the number of TGCT diagnoses by month of diagnosis, season, half year and histopathological subgroups.

Groups	Month	Cases ^1^ 2008–2018 Total (%)	Rate Ratio (95% CI) ^2^	*p*-Value	*P_interaction_*
Per month analysis					
January		349 (9.7)	1.14 (0.97 to 1.34)	0.123	-
February		312 (8.6)	1.11 (0.94 to 1.32)	0.203	
March		319 (8.8)	1.04 (0.88 to 1.23)	0.650	
April		299 (8.3)	1.01 (0.85 to 1.19)	0.941	
May		307 (8.5)	1.00 (Ref)		
June		275 (7.6)	0.93 (0.78 to 1.10)	0.380	
July		310 (8.6)	1.01 (0.85 to 1.19)	0.909	
August		260 (7.2)	0.85 (0.71 to 1.01)	0.064	
September		247 (6.8)	0.83 (0.70 to 0.99)	0.043	
October		314 (8.7)	1.02 (0.87 to 1.21)	0.791	
November		316 (8.7)	1.06 (0.90 to 1.26)	0.467	
December		307 (8.5)	1.00 (0.85 to 1.18)	1.000	
Per season analysis					
Winter	Jan–Mar	980 (27.1)	1.22 (1.11 to 1.35)	<0.001	-
Spring	Apr–Jun	881 (24.4)	1.09 (0.99 to 1.21)	0.0958	
Summer	Jul–Sep	817 (22.6)	1.00 (Ref)	-	
Fall	Oct–Dec	937 (25.9)	1.15 (1.04 to 1.27)	0.0078	
Per half year analysis					
Warm months	Apr–Sep	1698 (47.0)	1.00 (Ref)	-	-
Cold months	Oct–Mar	1917 (53.0)	1.13 (1.06 to 1.22)	<0.001	-
Stratification by time period					
2008 to 2013	Apr–Sep	842 (47.5)	1.00 (Ref)	-	0.559
	Oct–Mar	931 (52.5)	1.11 (1.00 to 1.23)	0.048	
2014 to 2018	Apr–Sep	856 (46.5)	1.00 (Ref)	-	
	Oct–Mar	986 (53.5)	1.16 (1.05 to 1.27)	0.0045	
Histology ^3^					
Pure Seminoma	Apr–Sep	1090 (45.6)	1.00 (Ref)	-	0.029
	Oct–Mar	1302 (54.4)	1.20 (1.10 to 1.31)	<0.001	
Non-Seminoma	Apr–Sep	428 (51.0)	1.00 (Ref)	-	
	Oct–Mar	412 (49.0)	0.97 (0.84 to 1.11)	0.64	
Mixed TGCT	Apr–Sep	57 (45.6)	1.00 (Ref)	-	
	Oct–Mar	68 (54.4)	1.20 (0.86 to 1.68)	0.30	
Age					
<40 years	Apr–Sep	971 (47.1)	1.00 (Ref)	-	0.852
	Oct–Mar	1090 (52.9)	1.13 (1.04 to 1.22)	0.0046	
≥40 years	Apr–Sep	727 (46.8)	1.00 (Ref)	-	
	Oct–Mar	827 (53.2)	1.13 (1.04 to 1.22)	0.0046	
Tumor spread					
Localized (T0 N0 M0)	Apr–Sep	1291 (46.7)	1.00 (Ref)	-	0.659
	Oct–Mar	1474 (53.3)	1.15 (1.06 to 1.24)	0.0013	
Generalized (T0–2, N1–4, M0; T3–4, N0–4, M0; Tx, N1–4, M0)	Apr–Sep	193 (49.2)	1.00 (Ref)	-	
	Oct–Mar	199 (50.8)	1.04 (0.85 to 1.26)	0.73	
Disseminated (T0–X, N0–X, M1)	Apr–Sep	80 (46.2)	1.00 (Ref)	-	
	Oct–Mar	93 (53.8)	1.17 (0.85 to 1.61)	0.35	

^1^ First day of each month excluded to avoid the wrong diagnosis dates due to imputation; ^2^ Corrected for the different length of the respective months; ^3^ 258 cases not included in analysis as definitive histological classification was missing. Abbreviations: TGCT = testicular germ cell tumor; T = tumor stage; N = lymph node status; M = metastases.

## Data Availability

The data presented in this study are available on request from the corresponding author. The data are not publicly available due to privacy and ethical reasons.
